# Pioneering Role of Nanopore Single-Molecule Sensing in Environmental and Food Surveillance

**DOI:** 10.3390/bios15010041

**Published:** 2025-01-13

**Authors:** Wenqiang Tian, Xu Wang, Yan Zhang, Ting Weng, Tlili Chaker, Xiaohan Chen, Qingke Kong, Deqiang Wang

**Affiliations:** 1 Chongqing Institute of Green and Intelligent Technology, Chinese Academy of Sciences, Chongqing 400714, China; 2 Chongqing School, University of Chinese Academy of Sciences, Chongqing 400714, China; 3 School of Pharmacy and Bioengineering, Chongqing University of Technology, Chongqing 400054, China; 4 College of Veterinary Medicine, Southwest University, Chongqing 400712, China

**Keywords:** nanopores, single-molecule sensing, metal ions, additives, polymers

## Abstract

In recent years, environmental and food safety have garnered substantial focus due to their intimate connection with human health. Numerous biosensors have been developed for identifying deleterious compounds; however, these biosensors reveal certain limitations. Nanopore sensors, featuring nano-scaled pore size, have demonstrated outstanding performance in terms of rapidity, sensitivity, and selectivity as a single-molecule technique for environmental and food surveillance. In this review, we present a comprehensive overview of nanopore applications in these two fields. To elucidate the pioneering roles of nanopores, analytes are categorized into three distinct groups, including metal ions, synthetic contaminants, and biotoxins. Moreover, a variety of strategies are involved, such as the coalescence with ligand probes, the implementation of chemical reactions, the functionalization of nanopores, etc. These scientific studies showcase the versatility and diversity of the nanopore technique, paving the way for further developments of nanopore technology in environmental and food safety.

## 1. Introduction

In recent years, the rapid pace of industrialization and human civilization gave rise to the emergence of numerous substances. These substances, including heavy metal ions, pesticides, plastics, additives, and biotoxins, are prevalently found in water, soil, and air, posing threats to environmental sustainability and food security. When tracing the course of historical events, numerous tragedies unfolded due to environmental and food hazards. For example, the year 1956 witnessed the reporting of 2248 cases and 1004 deaths in Japan due to mercury contamination in the environment and food [1]; in 1984, India was plagued by the catastrophic Bhopal gas leakage, which claimed 25,000 direct deaths and 550,000 indirect deaths [2]; and during the year of 2011, *Escherichia coli* O104:H4 caused an alarming number of over 830 cases of hemolytic uremic syndrome and 46 victims in Germany [3]. The issues of environment and food are of great importance for the survival of humanity; therefore, the surveillance in these areas establishes barriers to ensure protection, thus garnering widespread international focus. It should be noted that there is an intricate interplay between environmental pollution and food security. For example, the presence of pollutants in the environment will cause bioaccumulation, resulting in biomagnification within the human body. Given the intertwined nature of potential hazards in environmental monitoring and food security, forging a harmonious connection, it is justifiable to discuss these two fields concurrently.

To prevent the occurrence of tragedies, a multitude of diverse detection methods is implemented for environmental monitoring and food screening. For example, mass spectrometry (MS) serves not only as a reliable tool for the detection of metal ions but also can be coupled with chromatography techniques in the laboratory for the determination of ammonia nitrogen, ketones, and perfluorooctanesulfonic acid. Other proven techniques, including gas phase molecular absorption spectrometry, gas chromatography–infrared spectroscopy, and high-performance liquid chromatography, are also prevalently employed for different purposes in environmental and food surveillance [4,5,6]. However, these techniques have notable limitations such as cumbersome instruments, high costs, and requirements for skilled personnel, which restrict their wider usage. As the analysis of environmental and food samples will involve a diverse range of analytes, novel methods such as fluorescence spectrometry, electrochemical luminescence, and enzyme-linked immunosorbent assays are developed [7]. However, these methods still encounter challenges in terms of toxicity, sensitivity, and multi-component analysis, thereby struggling to fulfill the requirements of the surveillance.

Nowadays, single-molecule methods have exhibited robust performance in biosensing applications [8,9,10,11,12,13]. Nanopore technology, which monitors analytes at the single-molecule level, possesses the advantages of being label-free, sensitive, and portable [14,15,16]. After decades of development, nanopore technology has been demonstrated as a powerful tool for multiple analyte detections, including nucleic acids [17,18,19], peptides [20,21], and carbohydrates [22,23], thereby offering a novel solution for environmental and food surveillance. The principle of nanopore sensing is dependent on the interactions between analytes and the nanoscale pores, which induces distinctive electrical currents. These electrical currents offer multidimensional characteristics such as current blockage, the mean residence time, and event interval, which facilitate the identification of the nature, concentration, and geometry of samples [24,25,26]. Therefore, they serve as the fingerprints for analytes. With the development of nanopore technology, a wide range of nanopores have been discovered, including α-hemolysin (αHL) [27,28], *Mycobacterium smegmatis* porin A (MspA) [29,30], and aerolysin (AeL) [31,32]. By adopting diverse detection strategies tailored to the properties of each target analyte, the nanopore technique presents specific and flexible designs for disease diagnosis [33,34], homeland security monitoring [35,36], and pharmaceutical screening [37,38].

**Scheme 1:**
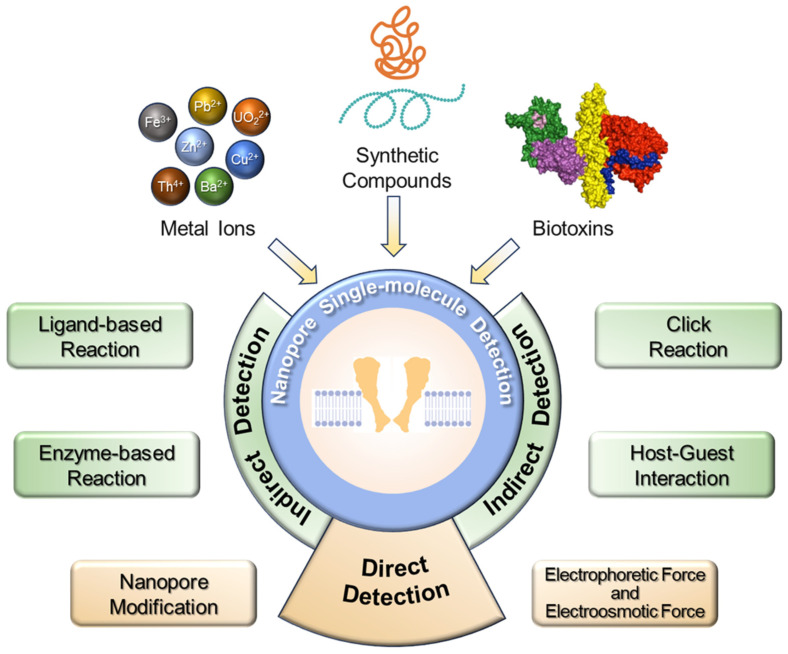
The schematic illustration of nanopore single-molecule detection in environmental and food surveillance employing different detection strategies.

In this review, we provide an overview of the remarkable accomplishments in nanopore single-molecule detection for environmental and food surveillance over recent years, highlighting its pioneering role. Given numerous analytes associated with environmental and food surveillance, they are classified into three categories based on their inherent characteristics, namely metal ions, synthetic pollutants, and biotoxins. Exemplary instances from each category, along with their specific detection strategies, are introduced to effectively showcase the pioneering role of nanopore technology. Due to the diverse nature of analytes, the detection strategies are categorized into direct and indirect methods. In direct detection, analytes are monitored in nanopores without the assistance of probes. Nanopore modification, electrophoretic force (EPF), and electroosmotic force (EOF) are effective methods for enhancing the measurements. For indirect detection, the presence of probes facilitates the occurrence of ligand-based reactions, catalysis-based reactions, enzyme-based reactions, host–guest interactions, etc., all of which integrate seamlessly with nanopores to achieve high sensitivity and selectivity (Scheme 1). Moreover, an in-depth analysis of the merits and demerits of each strategy, the potential challenges of nanopores, and future development are included in this review, offering valuable practical insights into this cutting-edge technology. In conclusion, this review comprehensively delineates the development of nanopores in environmental and food surveillance, thereby providing a systematic summarization of pertinent research.

## 2. Metal Ion Quantification

The quantification of metal ions bears immense significance in both environmental and food surveillance. Although certain metal ions, such as Fe^3+^ and Zn^2+^, are essential trace elements that confer benefits to living organisms, their excessive presence poses a grave threat to ecosystems and human health [39]. Moreover, it is desired to maintain a zero level of certain metal ions such as Pb^2+^ and Hg^2+^ in human blood, but these ions enter the human body through environmental and food routes, thereby posing serious risks to human health [40]. The presence of radioactive metal ions, such as UO_2_^2+^ and Th^4+^, will induce persistent genetic mutations, causing irreversible damage even in minuscule quantities [41]. The unbridled discharge of waste from industrial, agricultural, and daily activities leads to these pollutions, posing significant threats to the environment and food. The past few years have witnessed a multitude of noteworthy studies conducted by nanopores in the field of metal ion detection (Table 1) [42].

### 2.1. Heavy Metals

#### 2.1.1. Fe^3+^

**Figure 1:**
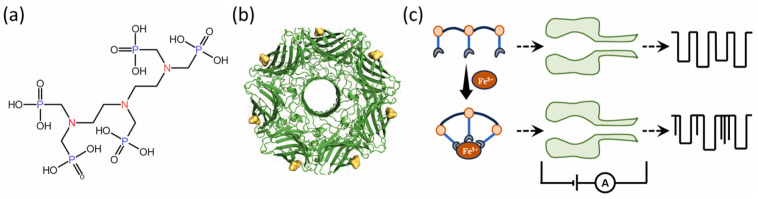
Nanopore sensing of Fe^3+^ with DTPMPA and a His-tagged α-HL M113K protein pore. Structures of (**a**) DTPMPA and (**b**) His-tagged M113K α-HL protein pore with the His-tag positions highlighted as gold spheres. (**c**) Schematic representation of the principle of Fe^3+^ detection. DTPMPA alone in the nanopore generated one major type of long-lived event. However, in the presence of Fe^3+^, a new type of event (attributed to the DTPMPA-Fe^3+^ complex) with a different dwell time was produced [45]. Copyright © 2024 Elsevier B.V. All rights reserved.

The entry of Fe^3+^ into the environment occurs through various routes, such as metal mining, industrial waste disposal, and pipe corrosion. While it poses little toxicity to humans and animals, it exhibits lethal effects on microorganisms, thereby impacting water quality and exerting a negative influence on the ecological environment [43]. Meanwhile, Fe^3+^ plays crucial roles in biological and metabolic processes, such as electron transfer reactions, oxygen transfer, and DNA synthesis. Hence, it is presented in nutrition products [44]. Recently, Guan’s group put forward a ligand–probe-based approach for quantifying Fe^3+^ in M113K α-hemolysin (αHL) nanopores, which was predicated on the chelation interaction between Fe^3+^ and Diethylenetriaminepenta (methylenephosphonic acid) (DTPMPA) (Figure 1) [45]. Owing to the negatively charged nature of DTPMPA, the anionic selectivity of nanopores was significantly enhanced by substituting methionine with lysine at position 113. In the absence of Fe^3+^, DTPMPA exclusively translocated through nanopores, inducing typical events with a mean residual current (I_b_) of 4.5 ± 1.2 pA and residence time (τ_off_) of 31.2 ± 4.9 ms. Conversely, in the presence of Fe^3+^, the formation of DTPMPA-Fe^3+^ complexes resulted in a distinct net charge, yielding a novel type of event characterized by I_b_ = 14.3 ± 0.8 pA and τ_off_ = 3.2 ± 0.5 ms. This sensor demonstrated a detection limit (LOD) of 0.28 μM for Fe^3+^. Although DTPMPA was acknowledged as a chelator for multiple metal ions, other metal ions exhibited distinct phenomena in simultaneous measurements (Zn^2+^: I_b_ = 5.3 ± 0.4 pA, τ_off_ = 23.6 ± 4.5 ms; Ni^2+^: I_b_ = 7.0 ± 1.2 pA, τ_off_ = 6.9 ± 0.3 ms; and Co^2+^: I_b_ = 6.5 ± 1.8 pA, τ_off_ = 91.3 ± 3.4 ms), which was accountable for the presence of the His-tags near the mushroom cap of nanopores. It should be noted that nanopores facilitated the discrimination of distinct interactions between targets and ligands, which enabled the simultaneous detection of multiple metal ions. In Asandei’s study, they found that Cu^2+^, Zn^2+^, Fe^3+^, and Al^3+^ presented various interactions with amyloid-β_1–16_ peptides [46].

#### 2.1.2. Cu^2+^

**Figure 2:**
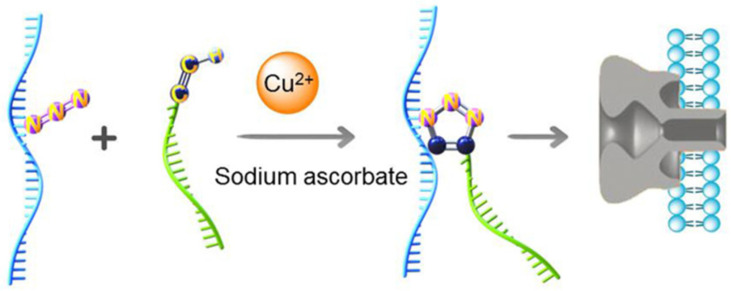
Schematics of the nanopore assay used for Cu^2+^ detection and real-time click reaction monitoring [47]. Copyright © 2019 American Chemical Society.

Metal ions potentially function as catalysts in chemical reactions, offering an innovative approach to their detections. For example, Liu L. et al. integrated click reactions into nanopore sensing to quantify Cu^2+^ [47]. Copper is an essential trace element in human physiology; however, excessive intake will result in gastrointestinal dysfunction, kidney damage, and neurodegenerative disorders [48,49]. As illustrated in Figure 2, the scheme comprised three steps as follows: 1. the reducing agent facilitated the conversion of Cu^2+^ to Cu^+^; 2. Cu^+^ activated the click reaction between two distinct types of ssDNA segments; and 3. nanopore analysis. The presence of Cu^2+^ facilitated the formation of forked DNA through the cycloaddition of azides and alkynes, which induced events with a current blockage (I_b_/I_0_) of 85% and a dwell time (τ_off_) of 1.19 ± 0.12 ms. Conversely, in the absence of Cu^2+^, no click reaction occurred and thus the events of ssDNA segments were observed (I_b_/I_o_ = 30% and τ_off_ = 0.16 ± 0.01 ms). Given that other metal ions (Al^3+^, Fe^3+^, Cr^3+^, Cd^2+^, Mg^2+^, Zn^2+^, Ca^2+^, Fe^2+^, Mn^2+^, Co^2+^, K^+^, and Na^+^) failed to activate click chemistry within this context, this sensor demonstrated exceptional selectivity with the LOD reaching 67 pM. In another study, Zhang Z. et al. opted to graft ferrocene⊂cucurbit[7]uril onto DNA segments by click reaction, enabling the quantification of Cu^2+^ as well [50]. It is worth mentioning that the click reaction enhanced the morphology of probes, which significantly improved the resolution of typical events in nanopores.

#### 2.1.3. Pb^2+^ and Zn^2+^

**Figure 3:**
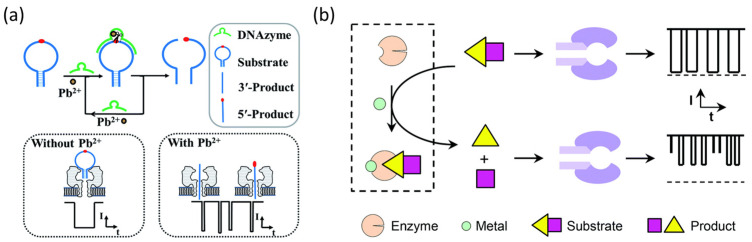
(**a**) Schematic illustration of the proposed label-free DNAzyme-based nanopore biosensor for Pb^2+^ detection. The target Pb^2+^ can trigger the DNAzyme catalysis cleavage reaction and release ssDNA products. Copyright © The Royal Society of Chemistry 2016. (**b**) Schematic representation of the enzymatic reaction-based nanopore detection of metal ions; without the target metal ions, the enzyme is inactive so that the current modulations are caused only by the peptide substrate, and with the analyte in the solution, the enzyme is activated and the cleavage of the peptide substrate by its corresponding enzyme produces new types of blockage events [51,52]. Copyright © The Royal Society of Chemistry 2019.

It is worth noting that specific metal ions activate certain enzymes, which could be utilized to identify metal ions in nanopores as well. For example, Liu G. et al. introduced a DNAzyme-based approach to monitor Pb^2+^ [51], whereas Roozbahani G. M. et al. utilized the hydrolysis of a metalloenzyme to detect Zn^2+^ [52]. The presence of Pb^2+^ or Zn^2+^ in water and soil is a common occurrence, resulting in bioaccumulation in organisms. The detection of Pb^2+^ involved the utilization of a hairpin DNA-RNA chimera as the substrate, which could be enzymatically cleaved by DNAzyme into two individual single-stranded DNA fragments (Figure 3a). The quantification of Pb^2+^ could be achieved by characterizing the typical events of hairpins (I/I_0_ = 74% and τ_off_ = 25 ms) and ssDNA (I/I_0_ = 87% and τ_off_ = 0.5 ms). This sensor demonstrated remarkable selectivity towards Ni^2+^, Mn^2+^, Zn^2+^, Ca^2+^, Mg^2+^, and NH_4_^+^ with high sensitivity (LOD = 3.48 nM). In contrast to Pb^2+^ detection, a disintegrin and metalloproteinase 17 (ADAM17) was employed for Zn^2+^ detection (Figure 3b). ADAM17 is a Zn^2+^-dependent enzyme that degrades the peptide substrate (LAQAVRSSSARLVFF) into two shorter peptide fragments (LAQAV and RSSSARLVFF). In the absence of Zn^2+^, the substrates exhibited typical events with I_b_/I_0_ of 27.9 ± 0.1% and τ_off_ of 3.00 ± 0.15 ms. Conversely, in the presence of Zn^2+^, the characteristic events of enzymatic fragments were observed (I_b_/I_0_: 42.4 ± 0.1% and τ_off_ = 0.78 ± 0.15 ms). This sensor exhibited no response towards Ni^2+^, Co^2+^, Hg^2+^, Cu^2+^, and Cd^2+^ when testing together with Zn^2+^. With a recording time of 10 min, its LOD reached 100 nM. The catalytic reaction of enzymes offered advantages in terms of high efficiency, selectivity, and sensitivity. When integrated with nanopores, this approach held promise as an alternative method for metal ion detection and expanded the applications of nanopores. The enzyme-catalyzed detection strategy described herein is applicable to a diverse range of other metal detections, such as Cd^2+^, Hg^2+^, and Cu^2+^ [53,54].

### 2.2. Inner Transition Metals

**Figure 4:**
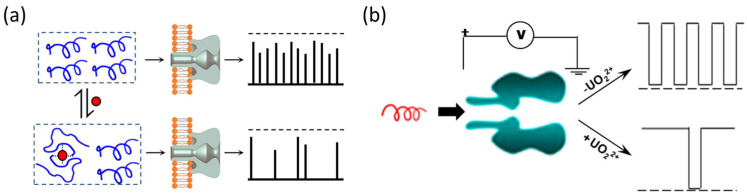
Schematic illustration of (**a**) Th^4+^ and (**b**) UO_2_^2+^ in nanopores [56,57]. Copyright © 2018 American Chemical Society and 2017 American Chemical Society.

Uranium and thorium are toxic chemical elements. The presence of these radioactive metals in food and water results in accumulation in the human body, thereby increasing the risk of poisoning and cancer [55]. In our previous study, we showed the application of peptides as probes for the selective detection of Th^4+^ and UO_2_^2+^, respectively (Figure 4). In the detection of Th^4+^, a 12-mer peptide (YEVHHQKDDPDD) was employed as the molecular probe, which interacted with Th^4+^ through chelation/coordination. In the absence of Th^4+^, the corresponding probes generated their characteristic events at high event frequencies, while in the presence of Th^4+^, a reduction in event counts was observed due to the conformational changes in the probes. Moreover, the sensor was measured using different buffer solutions with varying pH values. Notably, the performance of peptide-based nanopore sensors was optimized by adjusting the pH value, which gave rise to variations in the charges of peptides and ion–peptide complexes, as the net charge of peptides was pH-dependent. This peptide-based approach for Th^4+^ detection exhibited high selectivity (Ca^2+^, Zn^2+^, UO_2_^2+^, Pb^2+^, Cu^2+^, Ni^2+^, Hg^2+^, As^3+^, and Mg^2+^) with an LOD of 0.45 nM at pH 4.5 [56]. Similarly to Th^4+^, the detection of UO_2_^2+^ resulted in a comparable phenomenon by employing a peptide named HH14 as the probe [57]. This sensor demonstrated excellent selectivity (Ni^2+^, Cu^2+^, Zn^2+^, Cd^2+^, Pb^2+^, Hg^2+^, Th^4+^, Ca^2+^, and Mg^2+^) and sensitivity (LOD = 10 nM), which was further explored for water sample analysis and yielded convincible results. The aforementioned studies demonstrated that peptides were effective ligands for metal ion detection in nanopores. The sensor was impacted by the variations in the net charge of free peptides and ion–peptide complexes. Therefore, it is possible to logically predict the optimal pH buffer solution for nanopore sensing based on the composition of the peptide ligand.

### 2.3. Alkaline Earth Metals

Barium is abundant in the Earth’s crust, yet it has not been recognized as a necessary biological role in humans [58]. The main route of barium uptake in humans is from food and drinking water. Therefore, the screening of barium in environmental and food surveillance is crucial. The aptamer-based approach, which offers significant advantages in terms of high selectivity and cost-effectiveness, has been widely employed in nanopores to identify metal ions [59,60]. Therefore, Yang C. et al. developed a DNA probe by incorporating a 30-nucleotide tail at both ends of PS2.M (5′-GTAAGTAAAGCGAATTGG-3′) to specifically detect Ba^2+^ [61]. This probe was found to form a G-quadruplex structure with Ba^2+^. Consequently, without Ba^2+^, the probes generated rapid events (~0.5 ms) during their translocation through αHL, whereas in the presence of Ba^2+^, events with a τ_off_ value of 14.5 ± 1.1 ms were observed. This sensor demonstrated an LOD of 0.8 nM using an asymmetric salt buffer solution. Furthermore, its selectivity was assessed in the presence of Pb^2+^, K^+^, Na^+^, NH_4_^+^, Ca^2+^, Mg^2+^, Li^+^, Zn^2+^, Cd^2+^, Cu^2+^, Cr^3+^, Fe^3+^, and Hg^2+^. Although this probe exhibited binding affinity to Pb^2+^, the Pb^2+^–probe complexes induced events with a distinct dwell time (99.7 ± 1.1 ms). Despite some aptamers displaying selectivity to multiple targets, the structure variations between aptamers and targets could be discerned by nanopores.

**Table 1:** Summarized list of metal ion quantification and their detection strategies.

Targets	Detection Strategies		Nanopores	Probes	LOD	Ref.
Fe^3+^	Indirect Detection	Ligand-based Reaction	αHL (M113K)_7_	Organic molecule (DTPMPA)	0.28 μM	[45]
Cu^2+^	Indirect Detection	Click Reaction	αHL	DNA	67 pM	[47]
Pb^2+^	Indirect Detection	Enzyme-based Reaction	αHL	DNA	3.48 nM	[51]
Zn^2+^	Indirect Detection	Enzyme-based Reaction	αHL	Peptide	100 nM	[52]
Th^4+^	Indirect Detection	Ligand-based Reaction	αHL (M113K)_7_	Peptide	0.45 nM	[56]
UO_2_^2+^	Indirect Detection	Ligand-based Reaction	αHL (M113K)_7_	Peptide	10 nM	[57]
Ba^2+^	Indirect Detection	Ligand-based Reaction	αHL	DNA	0.8 nM	[61]

## 3. Synthetic Compound Identification

The escalating demands of lifestyle lead to a substantial augmentation in efficiency and convenience through the usage of synthetic compounds. However, their usages introduce a wide range of perils in environmental protection and food sources. For example, pesticides contribute to bountiful crop yields whilst simultaneously leaving behind residues on crops and causing ecological damage; plastic products and organic coatings offer convenience in daily life but disperse into the environment; and additives enhance the flavor and the nutrient contents at the expense of potential risks to human health. Nanopores exhibit high selectivity towards them and their analogs. In this section, we present several representative examples of nanopore-based synthetic compound identification (Table 2).

### 3.1. Pesticides

#### 3.1.1. Omethoate

**Figure 5:**
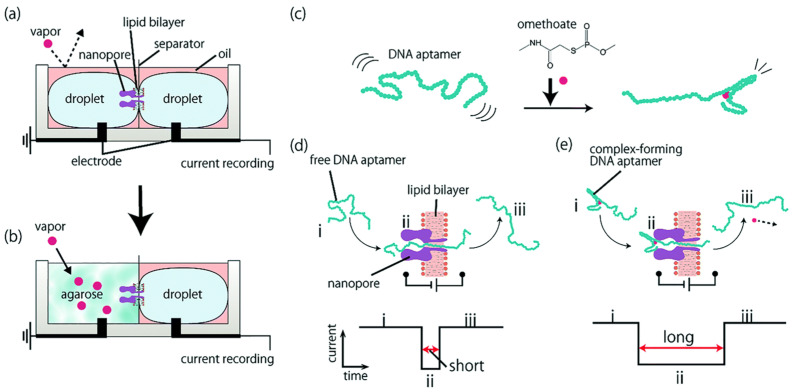
Schematic of the VOC nanopore sensor. (**a**) Formation of a lipid bilayer holding the nanopore by the general droplet contact method. (**b**) Modified agarose gel-based device for VOC absorption. (**c**) Formation of a rigid complex of the DNA aptamer and omethoate. (**d**) Free DNA aptamers translocate through the nanopore smoothly, whereas (**e**) the omethoate–DNA aptamer complexes are clogged due to the rigid secondary structure. Conceptual image of the DNA aptamer before (i), during (ii), and after (iii) translocation through the nanopore [62]. Copyright © The Royal Society of Chemistry 2017.

Nanopores have demonstrated the ability to detect analytes in solutions. However, there remains a question regarding their capability to detect volatile organic compounds (VOCs). Omethoate, an organophosphorus pesticide, is primarily employed to control pests in various crops. However, its prolonged usage leads to water and soil contamination. Being classified as a type of VOC, long-term exposure to omethoate leads to irreversible health implications. To establish a nanopore-based gas sensor, Fujii S et al. integrated hydrogels and DNA aptamers with αHL nanopores [62]. The hydrogels functioned as absorbents for omethoate in the air, facilitating its dissolution in solutions, while DNA aptamers selectively bound to omethoate in solutions for nanopore analysis. As illustrated in Figure 5, the absence of omethoate resulted in the observation of short residence time events with blockage of over 80%, while the presence of omethoate led to the appearance of longer residence time events. For quantification, events with a duration shorter than 510 ms and blockage larger than 80% were classified as aptamers, whereas those surpassing the thresholds of 510 ms and 80% were categorized as aptamer–target complexes. This gas sensor demonstrated high selectivity to omethoate, with event frequencies of omethoate being tenfold higher than those of four other pesticides (dichlorvos, methamidophos, EPN, and acetamiprid). Moreover, the sensor exhibited a high sensitivity, demonstrating an LOD of 4.8 nM in solution and 100 ppb in vapor. It should be noted that nanopore sensing should be performed exclusively in aqueous conditions, which limits its applications. However, the utilization of hydrogels for converting gas phase compounds into solutions indeed overcomes this limitation and explores the potential applications of nanopores in gas detection. To our knowledge, the aforementioned study stands as the inaugural achievement in the development of nanopore-based gas sensors. The nanopore in gas detection could be improved by adapting highly selective hydrogels and molecular amplification probes. It is worth mentioning that hydrogels are fabricated for biomarker detection in nanopores [63].

#### 3.1.2. Paraquat

**Figure 6:**
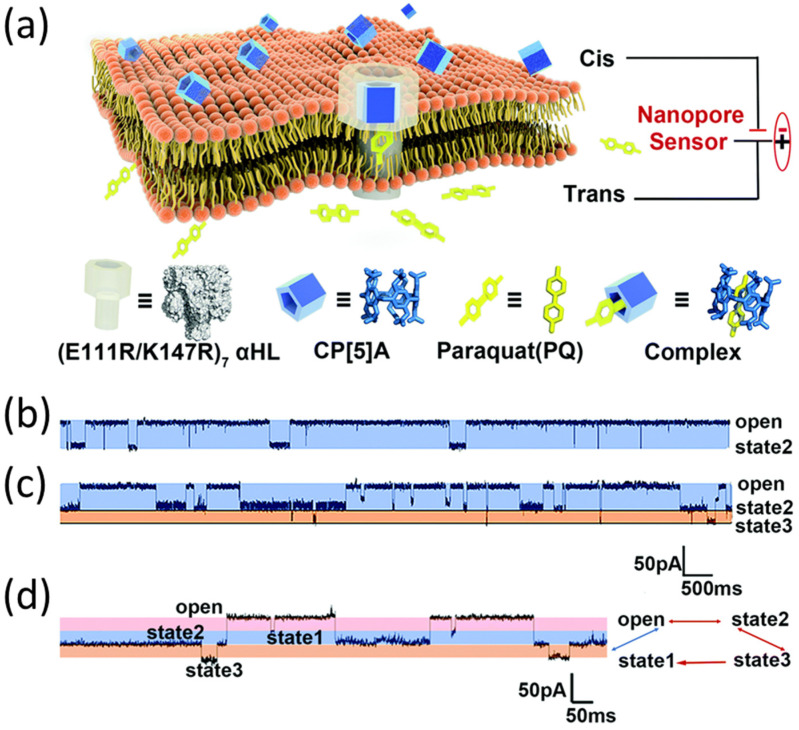
(**a**) A schematic of the new nanopore sensor for detecting paraquat (PQ), in which the CP[5]A acts as the recognition element introduced into the lumen of the (E111R/K147R)_7_ αHL nanopore; (**b**) the current reduction when CP[5]A was the sole molecule; (**c**) the current reduction when CP[5]A was added to the *cis* side and PQ was added to the *trans* side; (**d**) the enlarged view of the part of the current in (**c**) [64]. Copyright © the Partner Organisations 2021.

Paraquat (PQ) is another highly toxic pesticide that, due to its frequent usage, causes environmental pollution and food toxicity, thereby posing severe threats to ecosystems and human health. Due to the weak interactions between nanopores and PQ, host–guest interaction was integrated with nanopores. The host molecules employed in Jiang’s study were CP[5]A, which was a type of pillar[5]arene (P[5]A) with upper and lower rims modified with five carboxyl groups [64]. The presence of CP[5]A in (E111R/K147R)_7_ αHL nanopores would induce two types of events, namely State 1 (ΔI_0–1_/I_0_ = 0.17 ± 0.05 and τ_off1_ = 0.13 ± 0.04 ms) and State 2 (ΔI_0–2_/I_0_ = 0.33 ± 0.04 and τ_off2_ = 116.25 ± 0.05 ms), corresponding to random collisions and binding interaction, respectively. However, when both CP[5]A and PQ were present, a third type of event occurred, characterized by a current blockage of 0.48 ± 0.03 and a mean residence time of 9.91 ± 0.16 ms (Figure 6). These events exhibited dependence on PQ concentration and allowed for the determination of the LOD for PQ at 0.37 ppb, which was lower than the maximum residue limit of China’s National Food Safety Standard in food (62.5 ppb). In some cases, certain analytes may be too small or weak to elicit interactions with nanopores; thus, the incorporation of host–guest interactions allows for the construction of a sub-channel, facilitating the indirect interaction between nanopores and analytes.

### 3.2. Synthetic Materials

#### 3.2.1. Polyethylene

White pollution garbage poses a threat to environmental protection due to its difficulty in naturally degrading. Polyethylene (PE) is commonly utilized as a coating for paper cups, which possesses the potential to release microplastic particles at the nano or even sub-nano scale into aqueous solutions. Therefore, nanopores were employed for the measurement of various sizes of plastic particles [65]. Cho discovered that the electric currents of plastic particles in αHL nanopores were dependent on their sizes; the particles, which had a size comparable to the pore aperture, were trapped by nanopores, resulting in repeated opening and closing of the pore with short time intervals (~10 ms) due to particle vibration. The presence of slightly larger particles would insert into the pores, resulting in the occurrence of a blockage event; however, in the presence of molecules smaller than the pore size, the resistive pulse events appeared, with their magnitude being proportional to particle sizes. Moreover, the nanopore analysis revealed that the leaching of plastic particles from cups was temperature-dependent, where boiled samples yielded 1.8 times more particles compared to room-temperature samples. Nanopores enable not only the identification of analyte types but also the characterization of their dimensions.

#### 3.2.2. Per- and Polyfluoroalkyl Substances

**Figure 7:**
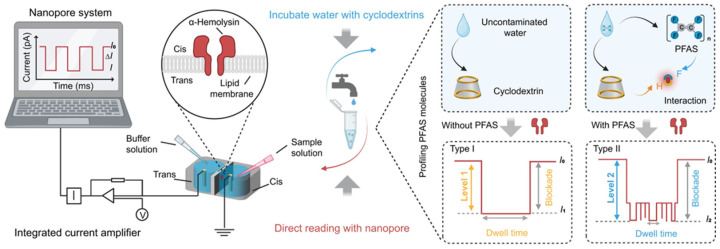
Schematic illustration of the experimental setup (**left**) and the CD-mediated nanopore assay workflow for profiling PFAS molecules (**right**). (**Left**) A nanopore system. An α-HL protein nanopore is inserted into a lipid bilayer membrane suspended across the 200 μm aperture on a Delrin wall that divides a chamber into two compartments, which are cis and trans. A command voltage is applied on the trans side, and the cis side is grounded. An integrated current amplifier is used for measuring transient current blockages caused by the translocation of sample molecules after adding the sample into the *cis* side. (**Right**) PFAS molecule profiling. The samples are achieved by incubating water possibly containing PFAS with CD, followed by the nanopore measurement of current blockage events for PFAS profiling and quantification [67]. Copyright © 2024 The Authors.

Per- and polyfluoroalkyl substances (PFAS) are another type of industrial material, which are prevalently utilized as waterproof and anti-fouling coatings for textiles [66]. After chronic usage, these substances are detected in water, air, and soil, thus being recognized as persistent and ubiquitous environmental pollutants. Inspired by the host–guest interactions, Liu’s group opted for cyclodextrins (CDs) to identify PFAS with αHL nanopores [67]. In their study, the interactions between PFAS and four types of CDs were investigated. Notably, HP-γ-CD, featuring additional hydroxypropyl groups on its side chains, exhibited the strongest affinity towards PFAS. As illustrated in Figure 7, in the absence of PFAS, HP-γ-CD generated characteristic events with an average I_b_/I_0_ of 0.863 ± 0.037 and τ_off_ of 0.313 ± 0.018 ms. However, the presence of PFOA or PFOS led to a shift in these events (HP-γ-CD-PFOA: I_b_/I_0_ = 0.712 ± 0.091 and τ_off_ = 16.9 ± 1.230 ms; HP-γ-CD-PFOS: I_b_/I_0_ = 0.667 ± 0.114 and τ_off_ = 29.27 ± 3.563 ms). Furthermore, a new type of subevent was observed (HP-γ-CD-PFOA: I_b_/I_0_ = 0.898 ± 0.124 and τ_off_ = 0.087 ± 0.008 ms; HP-γ-CD-PFOS: I_b_/I_0_= 0.806 ± 0.079 and τ_off_ = 0.087 ± 0.001 ms), which demonstrated the successful identification of PFOA and PFOS. The LODs for PFOA and PFOS were further determined to be 0.4 ppm and 2 ppm, respectively. By employing sample preconcentration, the LOD could be lowered to 400 ppt for both PFOA and PFOS. The measurement of the homologs of PFCA and PFSA families revealed that the current blockages of analytes were associated with the carbon chain length, thus demonstrating the outstanding capability of nanopores in identifying molecular structures. In their previous study, they employed host–guest interaction to detect PFOA bound to human serum albumin. Additionally, CD-based host–guest interactions exhibited remarkable capabilities in characterizing chiral molecules [68,69].

### 3.3. Additives

#### 3.3.1. Sweeteners

**Figure 8:**
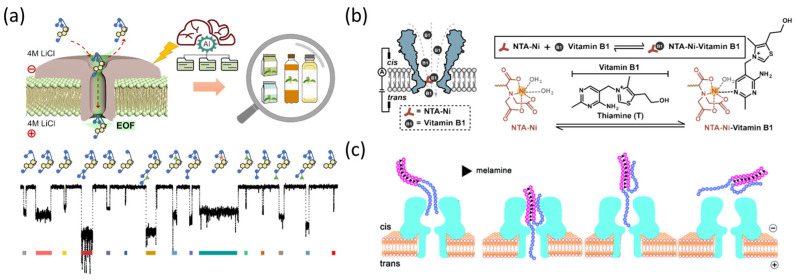
The detection of additives in nanopore sensors. (**a**) Schematic illustration of the test setup with AeL nanopores for the identification of 15 steviol glycosides, highlighting the fragments of ionic current traces associated with SGs [70]. Copyright © 2024 American Chemical Society. (**b**) The schematic diagram of vitamin B1 identification obtained by MspA-NTA-Ni nanopores. MspA-NTA-Ni is a hetero-octameric MspA modified with an NTA-Ni adapter at its pore constriction. Vitamin B1 reversibly binds to and dissociates from the NTA-Ni adapter, generating nanopore events. The pyrimidine moiety of vitamin B1 offers a coordination site to the bound Ni^2+^ of the NTA-Ni adapter [74]. Copyright © 2024 American Chemical Society. (**c**) Schematic diagram showing the interaction of the DNA–melamine hybrid with αHL nanopores [75]. Copyright © The Royal Society of Chemistry 2018.

In recent years, there has been an increasing emphasis on healthy diets, resulting in a growing preference for low-sugar or sugar-free food. Sweeteners are utilized to improve the flavor of these foods. Therefore, the detection of sweeteners has become increasingly important in food surveillance. Qing’s group reported the identification of 15 steviol glycosides (SGs) using EOF and EPF in AeL nanopores [70]. These 15 SGs shared the same core structure but had varying glycans. In their study, they discovered that the behaviors of SGs were voltage-dependent: when the voltage exceeded a certain threshold, the EOF would drive the translocation of SGs through nanopores; otherwise, the SGs would bind to the pore entrance due to the energy barrier. Although the presence of small structural differences in certain SGs, such as Ste and DuA, resulted in similar current blockages, the application of an increased voltage bias facilitated their identification, whose peaks were distinguishable at +160 mV. To further distinguish the nanopore signals of different SGs, Qing et al. collected individual blockage events of SGs and developed an end-to-end artificial intelligence model based on a deep learning algorithm. As a result, the resulting model achieved an overall accuracy of 93.6% (Figure 8a). The identification of various analytes using nanopores has been extensively documented; however, when combined with AI assistance, the nanopore sensor demonstrated enhanced accuracy and broader applications. AI is gradually employed in conjunction with nanopores for the identification of matrices [71,72]. Although nanopores have the ability to identify targets directly, chemical modification could enhance the selectivity of protein nanopores. For example, Huang’s group modified a single phenylboronic acid (PBA) adapter to MspA nanopores, enabling them to identify D-sorbitol and xylitol. In this manner, they also identified several types of cis-diols, including 1,2-diphenols, α-hydroxy acids, and saccharides [73].

#### 3.3.2. Vitamin Bs

Vitamin Bs are a group of small organic molecules that are essential exogenous nutrients for the human body. They were found to have extensive applications as nutritional supplements in health products, pharmaceuticals, and beverages. In nanopores, various strategies have been proposed to quantify vitamin Bs. For example, Maglia’s group utilized Cytolysin A (ClyA) nanopores to monitor the current fluctuations in the concentration of vitamin B1 and its binding protein [76]. Recently, a noteworthy study was conducted by Huang’s group, wherein they functionalized MspA nanopores to identify multiple vitamins Bs, including vitamin B1 (thiamine (T)), vitamin B3 (nicotinic acid (NA) and nicotinamide (NAM)), vitamin B5 (pantothenic acid (PA)), and vitamin B6 (pyridoxal (PL), pyridoxine (PN), and pyridoxamine (PM)) [74]. In Figure 8b, a nickel ion-bound nitrilotriacetic acid (NTA-Ni) adapter was modified at the 90th position of MspA nanopores, which presented different percentages of blockage of events (%I_b_ = (I_b_ − I_0_)/I_0_) towards vitamin Bs (T: 0.335 ± 0.010; NA: 0.566 ± 0.06; NAM: 0.434 ± 0.012; PA: 0.64 + 0.02; PL: 0.491 ± 0.005; PN: −0.559 ± 0.014; and PM: −0.304 ± 0.011, respectively). The negative values of PL and PN were attributed to spontaneous switching between analyte configurations. Although this sensor presented a millimolar level of LOD (T: 0.9 mM; NA: 0.02 mM; NAM: 0.02 mM; PA: 20 mM; PL: 2 mM; PN: 0.04 mM; and PM: 0.4 mM, respectively), it achieved the detection of vitamin Bs in cosmetic products and natural food with the assistance of a machine learning algorithm. The chemical modification offered protein nanopores enhanced selectivity to analytes. Beyond this study, this strategy has been applied for multiple purposes, such as saccharides [77], ribonucleotides [78], and amino acids [79].

#### 3.3.3. Melamine

Melamine (2,4,6-triamino-1,3,5-triazine) is primarily utilized in the production of resins, plastics, and glues. However, owing to its high nitrogen content, melamine was illegally added to milk, infant formula, and pet food as a means to improve the levels of proteins. The long-term and excessive intake of melamine leads to renal failure [80]. To address this issue, Sheng Y. et al. developed an aptamer-based approach utilizing αHL nanopores to detect melamine [75]. It was reported that melamine was capable of forming triple hydrogen bonds with thymine bases, thereby resulting in the formation of DNA–melamine hybrids. However, due to their large structure, these hybrids encountered hindered translocation, leading to prolonged current blockade events and reduced event frequencies (Figure 8c). To enhance the sensitivity of the melamine sensor, a series of probes were measured, resulting in the selection of the long T-containing probes with poly(dC) tails. The LOD was determined as 10 pM within a melamine concentration range of 10 pM to 100 nM, which was one of the lowest reported values to date. The presence of small organic molecules (cysteine, lactose, cyanuric acid, lysine, and vitamin C) and metal ions (Cu^2+^, Fe^3+^, Mg^2+^, Ca^2+^, and Zn^2+^) did not induce significant current blockades of the DNA probes in αHL nanopores. Notably, the sensor demonstrated practical application by detecting melamine spiked into milk. This detection strategy leveraged the specific interactions between substances and DNA probes. It offered valuable insights and guidance for designing highly selective nanopore detection sensors.

**Table 2:** Summarized list of synthetic compound identification and their detection strategies.

Targets	Detection Strategies		Nanopores	Probes	LOD	Ref.
Omethoate	Indirect Detection	Ligand-based Reaction	αHL	DNA aptamer	4.8 nM in solution and 100 ppb in vapor	[62]
Paraquat	Indirect Detection	Host–Guest Interaction	αHL (E111R/K147R)_7_	CP[5]A	0.37 ppb	[64]
Polyethylene	Direct Detection	EOF and EPF	αHL	None	None	[65]
PFAS	Indirect Detection	Host–Guest Interaction	αHL	HP-γ-CD	400 ppt	[67]
Steviol glycosides	Direct Detection	EOF and EPF	AeL	None	None	[70]
Cis-diols	Direct Detection	Nanopore Modification	MspA-PBA	None	None	[73]
Vitamin Bs	Direct Detection	Nanopore Modification	MspA-NTA-Ni	None	T: 0.9 mM, NA: 0.02 mM, NAM: 0.02 mM, PA: 20 mM, PL: 2 mM, PN: 0.04 mM, and PM: 0.4 mM	[74]
Melamine	Indirect Detection	Ligand-based Reaction	αHL	DNA	10 pM	[75]

## 4. Biotoxin Screening

Biotoxins are natural chemical substances produced by living organisms, which, under certain conditions, pose toxic effects on humans, animals, and the environment. The origins of these substances can be traced back to microorganisms, plants, or animals. They enter the ecological chain through contaminated food or environmental pollution, threatening public health and ecological balance. The detection of biotoxins is thus essential for maintaining ecological stability and protecting water and food safety. In this section, we summarize the screening of biotoxins using nanopore sensors, as detailed in Table 3.

### 4.1. Mycotoxin

**Figure 9:**
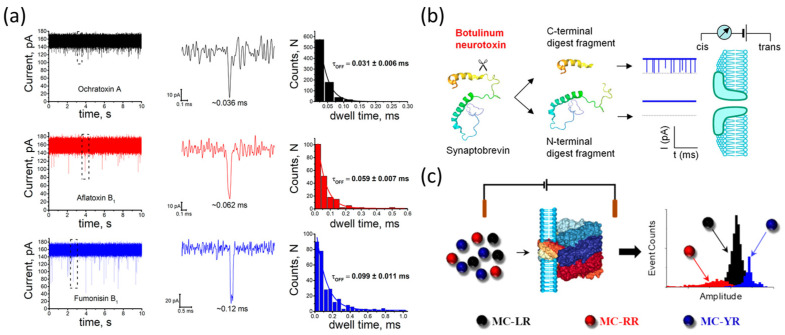
The detection of biotoxin in nanopore sensors. (**a**) Alphatoxin nanopore detection of mycotoxins. The typical ionic current, ionic current blockage events, and dwell time histograms of mycotoxin ochratoxin A (black), aflatoxin B_1_ (red), and fumonisin B_1_ (blue) in alphatoxin nanopores [82]. Copyright © 2023 by the authors. (**b**) Illustration of the principle for nanopore BoNT-B detection. After the cleavage of Lp-Sb2(1-93) by BoNT-B/Zn^2+^ (arrow), the short digested product Sb2(77-93)d would generate a distinct modulation of pore currents, as shown by downward transient blocks in the hypothetical trace, while the long product Lp-Sb2(1-76)d would not affect the nanopore current [84]. Copyright © 2014 American Chemical Society. (**c**) Schematic detection of cyclic heptapeptides microcystins in αHL nanopores [85]. Copyright © 2019 American Chemical Society.

Mycotoxins are metabolites produced by filamentous fungi, exhibiting toxic and carcinogenic properties [81]. Due to their widespread presence in food and feed products, Silva et al. conducted the identification of ochratoxin A (OTA), aflatoxin B_1_ (AFB_1_), and fumonisin B_1_ (FB_1_) in α-toxin nanopores [82]. In their study, these three mycotoxins were added into the *trans* side of the nanopore chamber, respectively, using 4 M KCl buffer solutions, where they exhibited distinct characteristics (OTA: I/I_0_ = 0.15 ± 0.001, τ_off_= 0.0311 ± 0.006 ms; AFB_1_: I/I_0_ = 0.326 ± 0.025, τ_off_ = 0.059 ± 0.007 ms; and FB_1_: I/I_0_ = 0.443 ± 0.025, τ_off_ = 0.0996 ± 0.011 ms). The coexistence of these mycotoxins did not yield any discrepancies in the results (OTA: I/I_0_ = 0.145 ± 0.021, τ_off_ = 0.028 ± 0.004 ms; AFB_1_: I/I_0_ = 0.364 ± 0.005, τ_off_ = 0.061 ± 0.007 ms; and FB_1_: I/I_0_ = 0.476 ± 0.002, τ_off_ = 0.095 ± 0.006 ms), indicating its potential to simultaneously detect multiple analytes. However, when measuring using a 1 M KCl buffer solution, there was a five-fold decrease in event frequencies compared to that at 4 M due to the salting-out effect. The salting-out effect was extensively applied in nanopores, leading to a significant enhancement in the performance of nanopores. The sensor exhibited a desirable LOD for OTA (1.0 nM), AFB_1_ (0.8 nM), and FB_1_ (0.45 nM), all of which were lower than the regulatory thresholds set for various foods (Figure 9a). Other methods could be employed for mycotoxin detection, such as Li T. et al.’s aptamer-based approach that achieved the detection of OTA with an LOD of 1.69 pM [83].

### 4.2. Bacterial Toxin

Some bacteria produce exotoxins during their growth, which causes severe human diseases. For example, *Clostridium botulinum* secretes botulinum neurotoxins (BoNTs). BoNTs are notorious as a biochemical weapon. However, traces of these toxins could be found in lightly preserved food items such as canned tuna, ham, and sausage. Due to their enzymatic function, Wang et al. developed the use of AeL nanopores for screening BoNT type B (BoNT-B) by exploiting its activity [84]. In Figure 9b, a recombinant synaptic peptide (Lp-Sb2) was employed as the substrate. Since BoNT-B is a Zn^2+^-dependent enzyme, measurements were taken in nanopores with BoNT-B or Lp-Sb2 alone or combined with Zn^2+^, resulting in no events observed. However, when BoNT-B, Lp-Sb2, and Zn^2+^ were present together, two types of events were observed with current values of I_r_ = 8.2 ± 0.09 pA and I_r_ = 26.8 ± 1.1 pA, respectively, indicating the occurrence of a hydrolytic reaction. Furthermore, investigations revealed that this approach could be applied to diluted serum samples. This enzyme-based approach mentioned above has also been utilized in the development of biomarker sensors [86,87]. Given that analytes possess their biofunctions, their bioactivity offers a novel perspective to sensor design.

### 4.3. Marine Toxin

Microcystins (MCs), whose general structures are cyclo(-D-Ala1-L-X2-D-erythro-β-methylAsp(iso-linkage)3-L-Z4-Adda5-D-Glu(iso-linkage)6-N-methyldehydro-Ala7), contaminate water and upset the balance of ecology, posing a threat to human and animals [88]. The maximum allowable concentration of these toxins in drinking water, as determined by the World Health Organization, is 1 μg/L. The MC variants were successfully detected by Zhou and Junior in αHL nanopores under varying experimental conditions, respectively [85,89]. MC-RR, MC-LR, and MC-YR are three distinct types of MCs that exhibit variations in L-amino acid composition. Since MC molecules are too large to translocate through αHL nanopores, collision events were collected. In Zhou’s study, these three molecules were measured using 1 M NaCl buffer solution and exhibited characteristics (MC-RR: I/I_0_ = −13.11 ± 0.10 pA, τ_off_ = 0.37 ± 0.04 ms; MC-LR: I/I_0_ = −8.66 ± 0.02 pA, τ_off_ = 1.02 ± 0.08 ms; and MC-YR: I/I_0_ = −5.78 ± 0.04 pA, τ_off_ = 2.08 ± 0.13 ms). Given that MCs are cyclic heptapeptides, enzyme reactions could be employed to generate linear analogs, thereby offering a novel approach to MC identification (Figure 9c). Due to the varying charges of MC-LR, MC-RR, and MC-YR, Junior et al. utilized 4 M LiCl buffer solutions to shield the charges of the stem region of the nanopores. As a result, the residence time of MC-RR decreased while that of MC-LR increased. It should be noted that the electrostatic interaction significantly influenced the performance of nanopores. In another study, our group reported the detection of MC-LR by an aptamer-based approach in solid-state nanopores [90].

**Table 3:** Summarized list of biotoxin screening and their detection strategies.

Targets	Detection Strategies		Nanopores	Probes	LOD	Ref.
OTA, AFB1, FB1	Direct Detection	EOF and EPF	αHL	None	OTA: 1.0 nM, AFB_1_: 0.8 nM, FB_1_: 0.45 nM	[82]
BoNT-B	Direct Detection	Enzyme-based Reaction	αHL	Peptide (Lp-Sb2)	Subnanomolar sensitivity	[84]
MCs	Direct Detection	EOF and EPF	αHL	None	None	[85,89]

## 5. Challenges and Future Perspectives

Despite its significant performance, nanopore technology still encounters some challenges. For example, multi-component analysis is anticipated in real sample detection. It is of significance that nanopore strategies focus on the detection of multiple analytes with high selectivity, which could be addressed by introducing aptamers, adaptors, and functional groups [91,92]. Another challenge that nanopores face is their inherent dimensional constraints, which limit the direct detection of certain analytes or reaction products. Therefore, it is desirable to design nanopores with comparable sizes for the detection of various analytes [93,94]. It should be noted that the single-molecule junctions have been demonstrated as an advanced biosensor [95]. Therefore, combining the mechanically controllable break junction technique with a nanopore may offer a strategy to overcome this challenge by providing an adjustable nanopore. Moreover, the identification of nanopore technology relies on variations in electrical events. However, due to the presence of structurally similar homologs among analytes, there may be overlap or minimal differences in events. Although experimental conditions such as pH, salt concentrations, and voltage biases could partially facilitate the distinction of these analytes, the processing of data analysis by incorporating multi-dimensional parameters is imperative to achieve higher accuracy [96]. It should be noted that due to the large volume of data and the blurred boundary between characteristics, manual data analysis may yield inaccurate results, especially in multi-component analysis with similar characteristics. Therefore, employing machine learning algorithms such as K-nearest neighbor, random forest, and naive Bayes to build analyte fingerprint libraries for the measurements of unknown samples improves the reliability of nanopore technology [79,97]. Additionally, to achieve practical and commercial applications of nanopore technology in environmental and food surveillance, it is challenging to develop integrated instruments similar to the Oxford Nanopore MinION, which are capable of automated device control and signal processing.

## 6. Conclusions

In conclusion, nanopore single-molecule detection exhibits high sensitivity and selectivity in the identification of diverse analytes such as metal ions, synthetic compounds, and biological toxins within environmental and food surveillance. This capability not only promotes environmental protection and pollution management, encompassing the monitoring of various pollutants in water and soil, but also contributes to ensuring food safety, such as the detection of biological toxins and pesticide residues in food. Although some analytes fail to induce considerable interactions with nanopores, different strategies can be tailored based on the physical and chemical properties of the analytes. Aptamer/peptide-based reactions, catalysis-based reactions, enzymatic reactions, host–guest interactions, and functional group modifications enhance the selectivity of nanopore detection, rendering their immunity to matrix impurities. Moreover, the implementation of sample pretreatment methods, such as sample preconcentration and hydrogel absorption, also improves the scenario of nanopores. When combined with machine learning techniques, nanopore technology has the potential to achieve a highly accurate detection of multiple components. Given these characteristics, with the advancement of nanopore technology, it is anticipated that nanopores play an irreplaceable role in the fields of environmental and food surveillance.

## Data Availability

No data were used for the research described in the article.
